# *Magnolia officinalis* Rehder & E.H.Wilson. Bark Extract and Magnolol Alleviate Allergic Rhinitis via Modulating NF-κB/MAPK Signaling

**DOI:** 10.3390/molecules31061009

**Published:** 2026-03-17

**Authors:** Leyuan Huang, Xu Zhou, Guanfeng He, Haixin Li, Xiaoying Chen, Jingwen Xu, Lei Zhou

**Affiliations:** School of Pharmacy, Guangdong Pharmaceutical University, Guangzhou 510006, China; 18877463934@163.com (L.H.); zx18319931770@163.com (X.Z.); 2112440294@stu.gdpu.edu.cn (G.H.); lhx235148@126.com (H.L.)

**Keywords:** allergic rhinitis, *Magnolia officinalis* Rehder & E.H.Wilson., magnolol, MAPK, NF-κB

## Abstract

*Magnolia officinalis* Rehder & E.H.Wilson. bark is famous as a traditional herbal medicine used in prescriptions for treating gastrointestinal discomfort, respiratory and inflammatory disorders. Magnolol, one of its principal bioactive constituents, exhibits potent anti-inflammatory and immunomodulatory properties. However, its therapeutic mechanisms in allergic rhinitis (AR) remain to be elucidated. In this study, the anti-allergic effects and molecular mechanisms of *M*. *officinalis* bark aqueous extract (MOAE) and magnolol were investigated using an ovalbumin (OVA)-induced AR mouse model. Nasal symptoms, histopathological alterations, and serum inflammatory mediators, including histamine and immunoglobulins (IgE, IgG1, IgG2a), were evaluated to assess efficacy. Both MOAE and magnolol significantly alleviated nasal rubbing and sneezing, reduced eosinophil infiltration and mucus hypersecretion, and improved tissue morphology in nasal and lung sections. Moreover, treatment markedly decreased serum levels of histamine and OVA-specific antibodies. Integrative network pharmacology, RNA sequencing, and molecular docking analyses revealed 33 co-regulated target genes mainly involved in the NF-κB and MAPK signaling pathways, suggesting that modulation of these pathways underlies the observed anti-inflammatory effects. These findings demonstrate that MOAE and magnolol exert protective effects against AR through the regulation of key inflammatory signaling cascades. This study provides modern pharmacological evidence supporting the traditional use of *M.* *officinalis* bark and highlights its potential as a natural therapeutic candidate for AR.

## 1. Introduction

Allergic rhinitis (AR) is a chronic inflammatory disease that is characterized by an immune response mediated by immunoglobulin E (IgE), with clinical manifestations including symptoms such as sneezing, nasal pruritus, rhinorrhea, and nasal congestion [1,2,3]. The worldwide prevalence of AR spans 10% to 50%, with children and young adults being disproportionately affected by a higher incidence rate [2,4]. As a consequence, patients suffer a considerable deterioration in their quality of life and there is a considerable rise in healthcare costs [2].

Although AR is a relatively less severe disease, its pathological process is actually quite complex and has not yet been fully elucidated. Its occurrence and progression are associated with immune pathways, including the degranulation of mast cell and the release of mediators such as histamine, Th2 polarization of helper T cells (Th2), and eosinophil infiltration, leading to the secretion of cytokines and pro-inflammatory mediators. Recent studies have also demonstrated that nociceptive sensory neurons in the nervous system are implicated in AR [2,5,6]. The activation of the nuclear factor-κB (NF-κB) pathway has been found to be closely associated with various inflammation-related diseases. Upon activation, NF-κB drives the transcription of pro-inflammatory cytokines, including tumor necrosis factor-α (TNF-α), interleukins (IL)-4, IL-5, and IL-13, which in turn recruit key immune cells such as eosinophils, Th2 lymphocytes, and basophils [7,8]. Likewise, the mitogen-activated protein kinase (MAPK) signaling pathway facilitates the escalation of inflammatory cascades by modulating gene transcription, cell proliferation, differentiation, and viability [9,10]. Together, these pathways contribute to both the early and late phases of AR, including nasal congestion, mucus production, and tissue remodeling. Therefore, modulation of these pathways represents a promising strategy for treating AR [11,12].

Current treatment strategies for AR focus mainly on allergen avoidance, drug-based treatments (such as corticosteroids, antihistamines, and decongestants), and allergen-specific immunotherapy [13,14]. Nevertheless, these existing treatments frequently fall short of delivering full symptom relief or are linked to adverse side effects, underscoring the necessity for novel therapeutic approaches. As the sole long-term intervention, immunotherapy demands extended treatment spanning multiple years, which limits its practicality for a large number of patients [15]. In light of these constraints, there is an urgent demand for alternative therapeutic approaches that are both effective and well-tolerated, especially those that can target the fundamental inflammatory mechanisms of AR.

**Figure 1:**
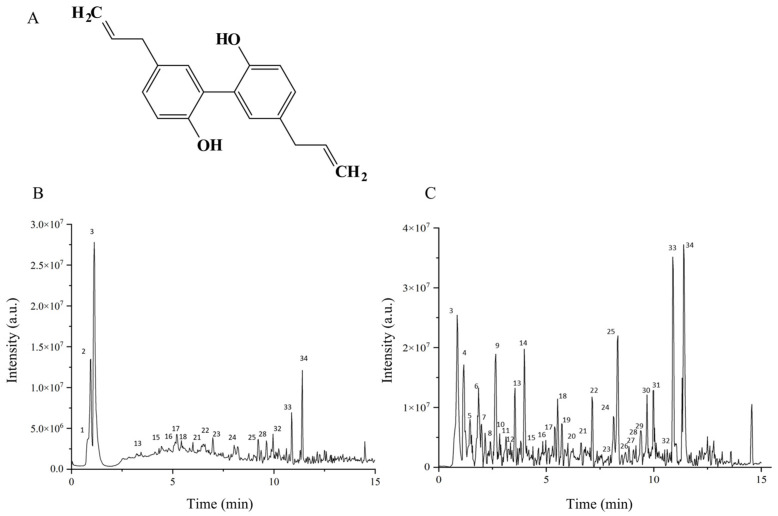
Chemical profiling of MOAE. (**A**) Chemical structure of Magnolol. (**B**) TIC peak diagram of positive departure mode MOAE sample. (**C**) TIC peak diagram of negative deviation mode MOAE sample.

*Magnolia officinalis* Rehder & E.H.Wilson. bark has been used for centuries due to its anti-inflammatory, antioxidant, and anxiolytic properties [16,17,18]. Researchers have discovered that dry flower buds of *M*. *officinalis* possess potential therapeutic effects on allergic diseases [19]. The active components of *M*. *officinalis* bark that have been extensively studied are two lignans: magnolol (Mag, Figure 1) and honokiol. Magnolol is recognized for its anti-inflammatory, antioxidant, and neuroprotective effects [20,21,22,23,24]. Recently, magnolol was found to alleviate AR via inhibition of ORAI1 and ANO1 channels [25]. Honokiol has been demonstrated to reduce symptoms of AR through the modulation of TMEM16A, TRPV1, and calcium signaling [26]. These suggest that *M*. *officinalis* bark may also possess similar AR-relieving effects. In addition, some traditional Chinese medicine preparations that include *M*. *officinalis* bark, for example, Chai-Pu-Tang, are known to reduce airway inflammation in disorders [27,28]. However, the alleviating effect of *M*. *officinalis* bark on AR and its potential mechanisms remain unclear.

Since magnolol has been demonstrated to possess therapeutic efficacy against AR in previous studies, this study simultaneously investigates both *M*. *officinalis* bark and magnolol to further explore their therapeutic efficacy and potential molecular mechanisms in treating AR. In the present study, we examined the chemical composition of MOAE and employed bioinformatics analysis to identify potential components and targets from this plant with therapeutic effects on AR. Additionally, we validate these findings in an AR mouse model, assessing the efficacy of MOAE and magnolol in reducing symptoms and pathological alterations. By using transcriptomic analysis and molecular biology techniques, we aim to uncover the underlying mechanisms through which *M*. *officinalis* bark exerts its anti-AR effects.

## 2. Results

### 2.1. Chemical Profiling of MOAE

An UPLC-Q-TOF-MS system was utilized for the purpose of analyzing the chemical composition of the MOAE under investigation. Altogether 34 components were detected through comparison of MS1 and MS2 data against an in-house database. Figure 1 showed the total ion chromatograms (TIC) in positive ion mode and negative ion mode. Details of the identified components of MOAE are presented in Table 1.

These constituents include 5 alkaloids (betaine, reticuline, magnoflorine, anonaine and liriodenine), 6 phenolic glycosides (such as acteoside and various magnolosides), 17 lignans and neolignans (such as syringaresinol, magnolignans A, C, D, Mag and honokiol), 5 phenolic aldehydes and derivatives (magnaldehydes B, D, E, randaiol and obovatol), along with one simple phenolic acid (caffeic acid) and two other compounds. The varied constituents of MOAE reflects its intricate chemical makeup.

### 2.2. Bioinformatics Analysis of MOAE in AR Treatment

**Figure 2:**
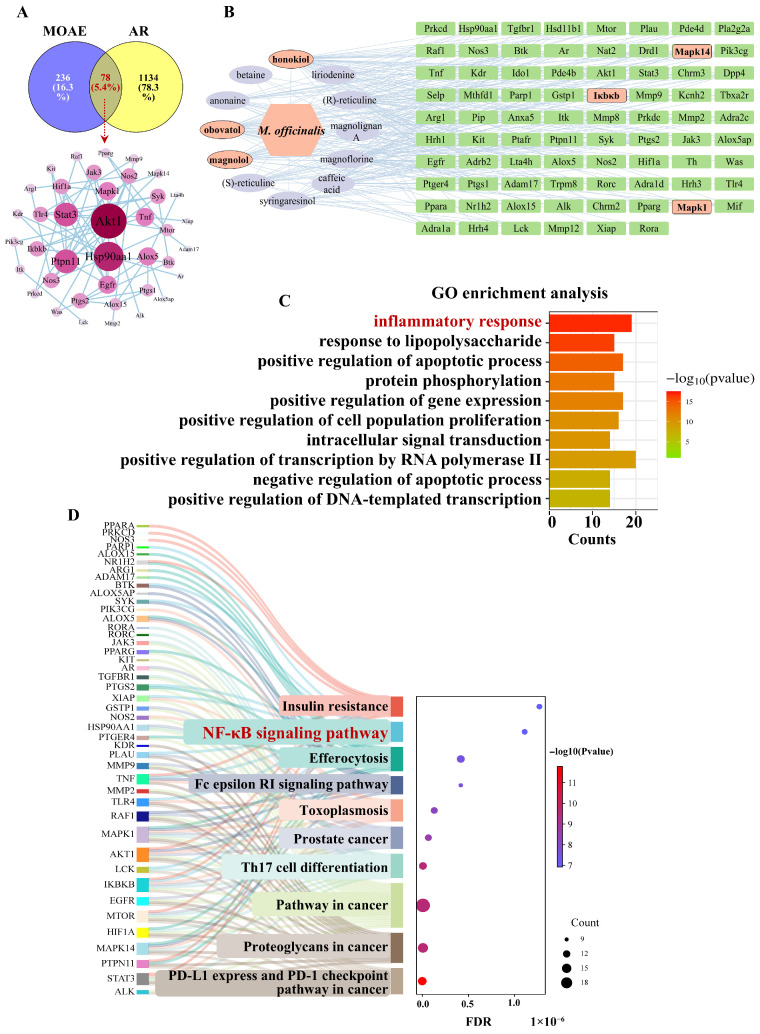
The results of bioinformatics analysis. (**A**) Venn diagram of intersection target of drugs and diseases and drug ingredient target network diagram. (**B**) Core target protein–protein interaction (PPI) network diagram. (**C**) The result chart of GO enrichment analysis. (**D**) Key pathway target network diagram.

A comparison between targets of active components identified in MOAE and those associated with AR revealed 78 overlapping targets, including Akt1, Hsp90aa1, Stat3, Mapk1, Ptpn1l, Ikbkb, Mapk14, Ptgs2 (Figure 2A). The key active ingredients in MOAE, such as honokiol, reticuline, obovatol, Mag, caffeic acid, and magnoflorine, interact with a wide range of targets, suggesting that they may serve as the primary pharmacological agents responsible for MOAE’s therapeutic effects (Figure 2B). Based on the analysis results, Mag was identified as one of the compounds with the strongest correlation to AR therapy. Consequently, we selected Mag for further pharmacodynamic validation and mechanism studies. In addition, Gene Ontology (GO) functional enrichment analysis was conducted using a screening criterion of false discovery rate (FDR) lower than 0.05, yielding a total of 695 GO entries. In terms of biological processes, MOAE primarily participates in the inflammatory responses, response to lipopolysaccharide and positive regulation of RNA polymerase II-mediated transcription (Figure 2C). Kyoto Encyclopedia of Genes and Genomes (KEGG) pathway enrichment analysis identified 131 relevant pathways overall. As indicated by the results of the pathway enrichment analysis, the therapeutic effects of MOAE on AR are primarily mediated through three signaling pathways, i.e., NF-κB, high-affinity immunoglobulin E Fc receptor (FcεRI) and Th17 cell differentiation (Figure 2D).

### 2.3. MOAE or Mag Relieves OVA-Induced Nasal Allergy Symptom

**Figure 3:**
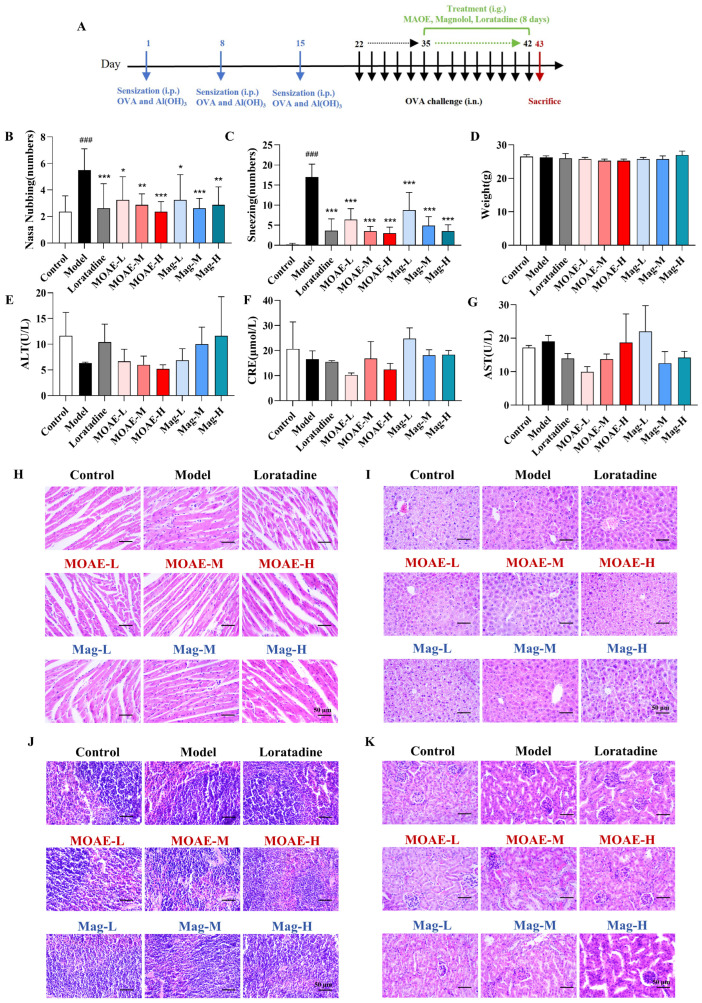
The effect of MOAE or Mag on AR mice. (**A**) The experimental process of the AR model establishment and administration. Frequency of (**B**) nasal rubbing and (**C**) sneezing in mice (*n* = 8). (**D**) The weight of the mice on the 42nd day (*n* = 4). (**E**) Serum ALT level (*n* = 3). (**F**) Serum CRE level (*n* = 3). (**G**) Serum AST level (*n* = 3). (**H**) Images of H&E staining of the heart (200×). Bars = 50 µm. (**I**) Images of H&E staining of the liver (200×). Bars = 50 µm. (**J**) Images of H&E staining of the spleen (200×). Bars = 50 µm. (**K**) Images of H&E staining of the kidney (200×). Bars = 50 µm. Data are presented as mean ± SD. ^###^ *p* < 0.001, compared with the Control group. * *p* < 0.05, ** *p* < 0.01, *** *p* < 0.001, compared with the Model group.

On the final day of the experiment, behavioral observations and recordings were immediately performed on the mice following intranasal OVA challenge (Figure 3A). As shown in Figure 3B,C, OVA-sensitized mice demonstrated a significantly elevated frequency of both sneezing and nasal rubbing in comparison to the control group. By contrast, MOAE or Mag administration for 8 days markedly attenuated these OVA-induced allergic symptoms. A comparable therapeutic effect was noted in the positive control group administered loratadine. As the experiment progressed, no statistically significant differences in mouse body weight were observed in relation to the model group on day 42 (Figure 3D). Additionally, there were no significant differences in the levels of serum alanine aminotransferase (ALT), aspartate aminotransferase (AST), or creatinine (CRE) across all groups (Figure 3E–G). Hematoxylin-eosin (H&E) staining of organ tissues revealed that MOAE or Mag did not exert any significant effects on the heart (Figure 3H), liver (Figure 3I), spleen (Figure 3J), or kidney (Figure 3K) when compared to control mice.

### 2.4. Effect of MOAE or Mag on the Production of Histamine and OVA-Specific Antibodies in Serum

**Figure 4:**
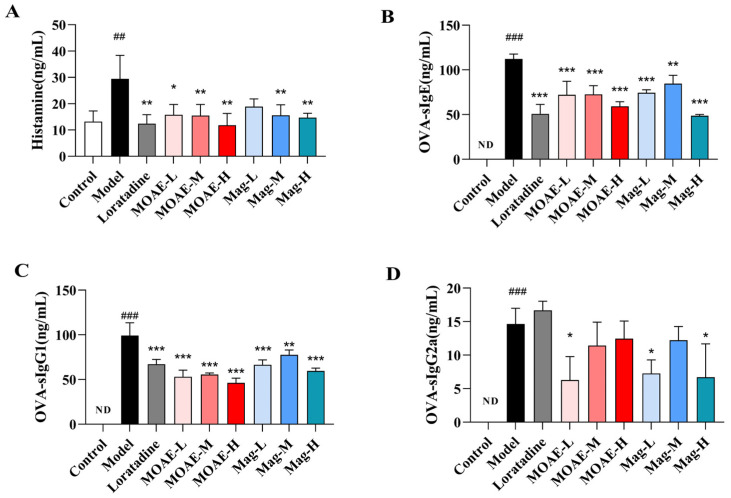
Effect of MOAE or Mag on the production of histamine and OVA-specific antibodies in serum. The levels of (**A**) histamine. (**B**) OVA-sIgE. (**C**) OVA-sIgG1. (**D**) OVA-sIg2a in the serum of mice (*n* = 3). Data are presented as mean ± SD. ^##^ *p* < 0.01, ^###^ *p* < 0.001, compared with the Control group. * *p* < 0.05, ** *p* < 0.01, *** *p* < 0.001, compared with the Model group. ND, not detected.

Histamine is a mediator released by mast cells, and its increased release is often detected during the onset of AR in patients. Suppressing histamine production and release can mitigate AR symptoms. To explore how MOAE or Mag affects histamine levels, serum histamine concentrations were quantified using ELISA. As shown in Figure 4A, OVA increased the serum histamine level, while MOAE or Mag significantly downregulated the histamine levels. The effect of MOAE-H was comparable to that of loratadine. Given the key role of immunoglobulins in mediating allergic and inflammatory responses, we further assessed serum OVA-specific IgE (sIgE), sIgG1, and sIgG2a in OVA-induced AR mice, molecules linked to B-cell immune responses regulated by helper T cell-derived cytokines. As shown in Figure 4B, OVA-induced mice exhibited a notable increase in OVA-specific IgE levels compared to the control group, while MOAE, Mag, or loratadine treatment decreased the levels of serum OVA-sIgE. A similar trend could be observed in the serum levels of OVA-sIgG1 (Figure 4C) and sIgG2a (Figure 4D). Collectively, these findings demonstrate that MOAE or Mag attenuate allergic responses by regulating serum immunoglobulin levels.

### 2.5. Effect of MOAE or Mag on Histopathological Alterations in Nasal Mucosa

**Figure 5:**
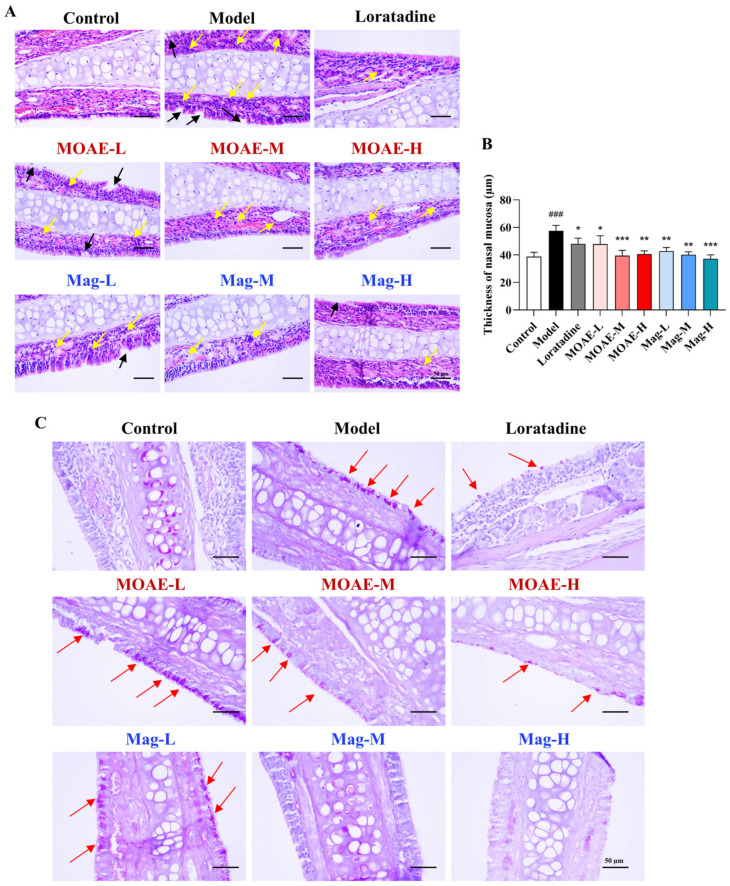
Effect of MOAE or Mag on the nasal mucosa thickness and goblet cell hyperplasia in the nasal mucosa. (**A**) Images of H&E staining of the nasal mucosa. Bars = 50 µm. (200×). (**B**) Thickness of nasal mucosa (*n* = 3). Data are presented as mean ± SD. ^###^ *p* < 0.001, compared with the Control group. * *p* < 0.05, ** *p* < 0.01, *** *p* < 0.001, compared with the Model group. (**C**) Images of PAS staining of the nasal mucosa (200×). Bars = 50 µm, goblet cells in red arrow.

Histological examination revealed that the nasal mucosa structure in the model group was disrupted, with local damage and shedding of the epithelial layer, and cilia were found lying down (Figure 5A, black arrow). Numerous inflammatory cells were diffusely infiltrated into the lamina propria (Figure 5A, yellow arrow). In contrast, nasal mucosal damage in the treatment groups was significantly reduced. The epithelial layer displayed a more organized structure, with epithelial shedding areas minimized and infiltration of inflammatory cells (e.g., eosinophils) notably decreased. Moreover, the extent of vascular dilation, congestion, and tissue edema was lessened. As shown in Figure 5A,B, MOAE, Mag, or loratadine treatment decreased the OVA- induced nasal mucosa thickening. The evaluation of goblet cell hyperplasia was conducted through the utilization of Periodic acid-Schiff (PAS) staining. In Figure 5C, an increase in the number of goblet cells could be observed in the presence of OVA induction. Conversely, treatment with MOAE, Mag, or loratadine led to a decrease in goblet cell hyperplasia (red arrow). These findings indicate that MOAE or Mag exerts a protective effect on the nasal mucosa, reducing inflammatory cell infiltration in the nasal mucosa of AR mice.

### 2.6. Impact of MOAE or Mag on Histopathological Alterations in Lung and Tracheal Tissues

**Figure 6:**
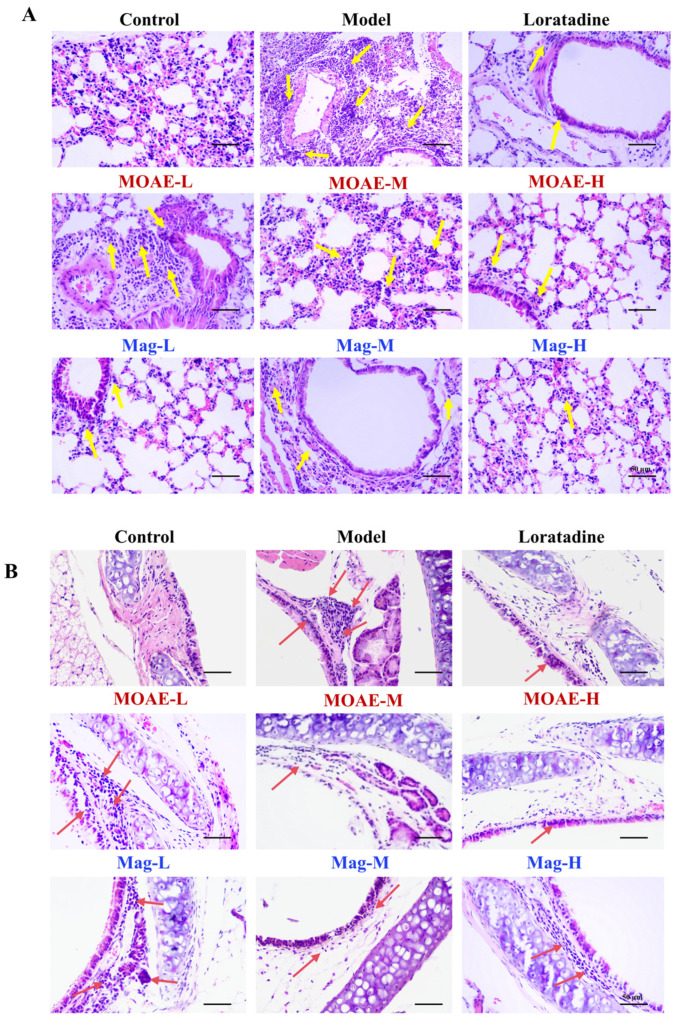
Impact of MOAE on histopathological alterations in lung and tracheal tissues. (**A**) Images of H&E staining of the lung. (**B**) Images of H&E staining of the trachea. Bars = 50 µm (200×).

Histopathological analysis of lung tissues revealed significant inflammatory changes in the OVA-challenged mice, showing excessive mucus accumulation in the bronchial lumens and pronounced inflammatory cell infiltration (Figure 6A, yellow arrow). Notably, both MOAE and Mag treatment substantially reduced these pathological alterations, with reduced mucus secretion and diminished inflammatory cell recruitment (Figure 6A, yellow arrow). Histopathological analysis of tracheal tissues further confirmed these findings. Both inflammatory cell infiltration and epithelial thickening were noted in the Model group—hallmark characteristics of allergic airway inflammation. Conversely, mice administered MOAE or Mag showed a marked decrease in inflammatory cell recruitment (Figure 6B, red arrow). These effects on the tracheal structure were consistent with the improvements observed in lung tissue H&E staining. Collectively, these findings indicate that MOAE or Mag diminish mucus accumulation, mitigate airway epithelial thickening, ameliorate airway remodeling, and lessen inflammatory infiltration in bronchial and lung tissues of OVA-induced AR mice.

### 2.7. Transcriptomic Analysis of MOAE or Mag in AR Mice

**Figure 7:**
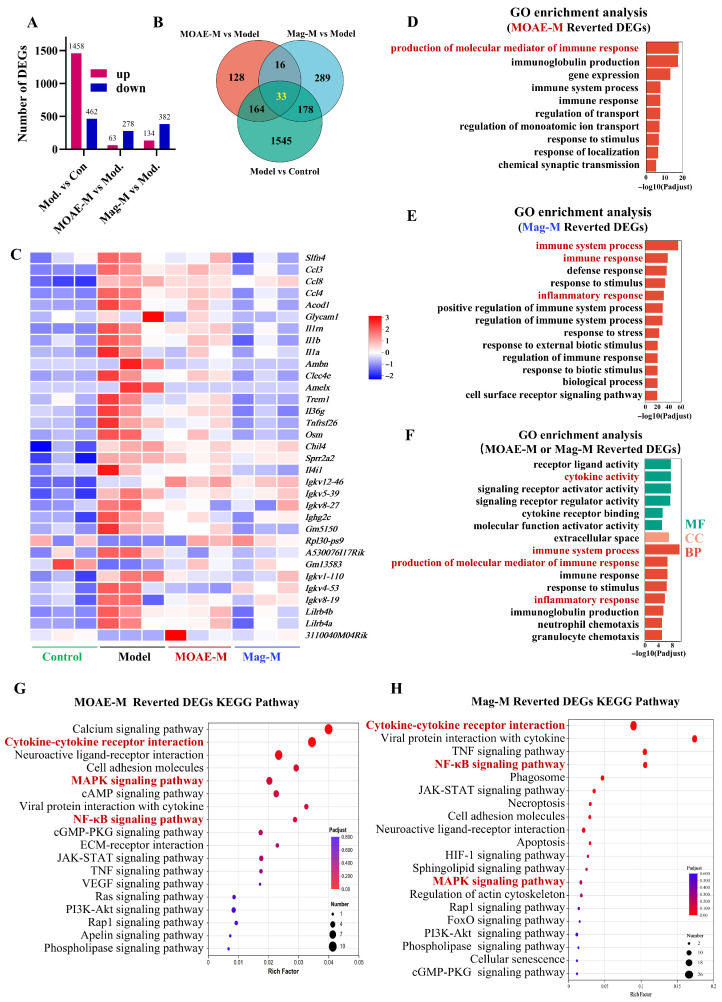
Transcriptomic altered with MOAE or Mag treatment in AR mice. (**A**) DEGs statistical chart of expression level differences. (**B**) Venn diagram of target gene set. (**C**) Cluster analysis heatmap of target gene set. GO enrichment analysis. (**D**) MOAE-M reverted DEGs. (**E**) Mag-M reverted DEGs. (**F**) MOAE-M or Mag-M reverted DEGs. KEGG enrichment analysis. (**G**) MOAE-M reverted DEGs. (**H**) Mag-M reverted DEGs.

To explore the molecular mechanisms driving the effects of MOAE and Mag in AR, we conducted RNA sequencing and analyzed differentially expressed genes (DEGs) via pairwise comparisons among the control, model, MOAE-M, and Mag-M groups. The results indicated that, in comparison with the control group, the Model group exhibited 1458 genes that demonstrated a significant increase in expression and 462 genes that exhibited a significant decrease in expression. Compared to the model group, the MOAE-M treatment group exhibited 63 upregulated and 278 downregulated genes. Similarly, the Mag-M treatment group displayed 134 upregulated and 382 downregulated genes (Figure 7A). These data indicate that both the AR mouse model and subsequent drug treatments triggered significant gene expression changes in the nasal mucosa. Further Venn diagram analysis identified 33 genes commonly regulated across the model, MOAE-M, and Mag-M groups, suggesting potential overlap in the therapeutic mechanisms of MOAE and Mag in AR (Figure 7B). To examine relationships among these 33 co-regulated genes, we performed cluster analysis. The application of a heatmap revealed distinct patterns of gene expression across groups. In this context, darker red indicates higher levels of expression, while darker blue indicates lower levels of expression. Gene expression trends were consistent within each group, and marked differences were observed between groups (Figure 7C). Notably, genes linked to inflammatory cell recruitment (e.g., Ccl3, Ccl4, Ccl8) were significantly upregulated in AR mice; Ccl8 showed a strong positive correlation with eosinophil counts and nasal tissue inflammation severity. Additionally, genes associated with mucus secretion, tissue remodeling, and acute inflammatory responses (Il-1β, Il-1α) were significantly elevated in AR mice. These genes were substantially downregulated after treatment with MOAE and Mag. In GO enrichment analysis, MOAE or Mag treatment was mainly involved in three processes, which were immune system processes, inflammatory response, and cytokine activity (Figure 7D–F). KEGG pathway analysis focuses on changes in signaling pathways. As shown in Figure 7G,H, after treatment with MOAE or Mag, the key target genes were enriched in pathways related to cytokine–cytokine receptor interactions, NF-κB and the MAPK pathway.

Overall, transcriptomic analysis indicates that MOAE and Mag exert therapeutic effects in AR by regulating 33 co-modulated genes. Their mechanisms of action seem to involve immune system processes, inflammatory responses, cytokine–cytokine receptor interactions, NF-κB, and the MAPK pathway.

### 2.8. Molecular Docking Verification of Key Hub Genes

**Figure 8:**
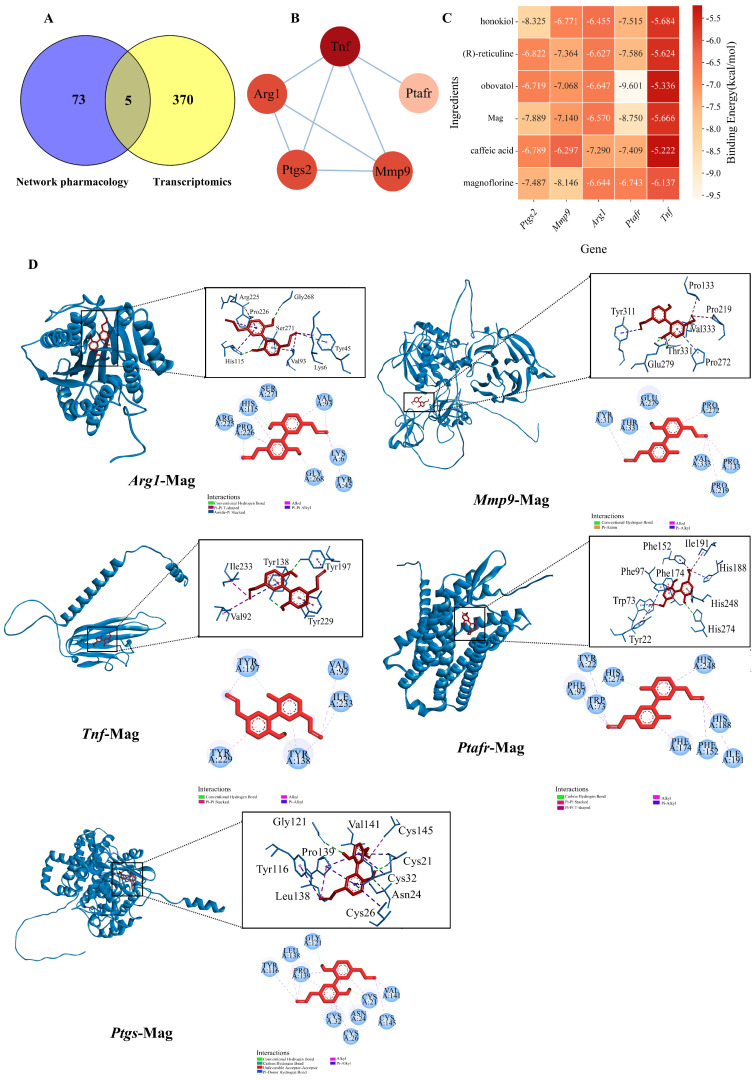
Molecular docking. (**A**) Effective targets from network pharmacology combined with DEGs from RNA-seq. (**B**) 5 key target genes PPI. (**C**) Molecular docking binding energy heatmap. (**D**) Visualization of molecular docking.

Cross-matching 78 validated targets in network pharmacology with 375 DEGs identified through RNA-seq analysis revealed five core overlapping target genes: Ptgs2, Mmp9, Arg1, Ptafr, and Tnf (Figure 8A,B). Among these, both Tnf and Ptgs2 directly participate in inflammatory reactions, while Mmp9 (regulated by Tnf) synergistically drives inflammation and tissue injury. Subsequently, we conducted molecular docking analyses between the six most highly screened active components and the aforementioned target genes to elucidate the pharmacophore of MOAE. The AutoDockVina tool (version 1.2.0) was employed to calculate the free binding energy between the active components and the hub target genes (Figure 8C). Negative binding affinity signifies potential receptor–ligand binding; specifically, values < −5 kcal/mol suggest strong interactions, with <−7 kcal/mol indicating particularly robust binding. This implies the five ligands can structurally stabilize and bind spontaneously to the Ptgs, Mmp9, Arg1, Ptafr, and Tnf receptors. Among them, Mag was further utilized for molecular docking simulation experiments with each hub target gene. As shown in Figure 8D, Mag had strong binding affinities: −7.889 kcal/mol with Ptgs2, −7.140 kcal/mol with Mmp9, −6.570 kcal/mol with Arg1, −8.750 kcal/mol with Ptafr, and −5.666 kcal/mol with Tnf.

### 2.9. Verification of Potential Hub Genes by qRT-PCR

**Figure 9:**
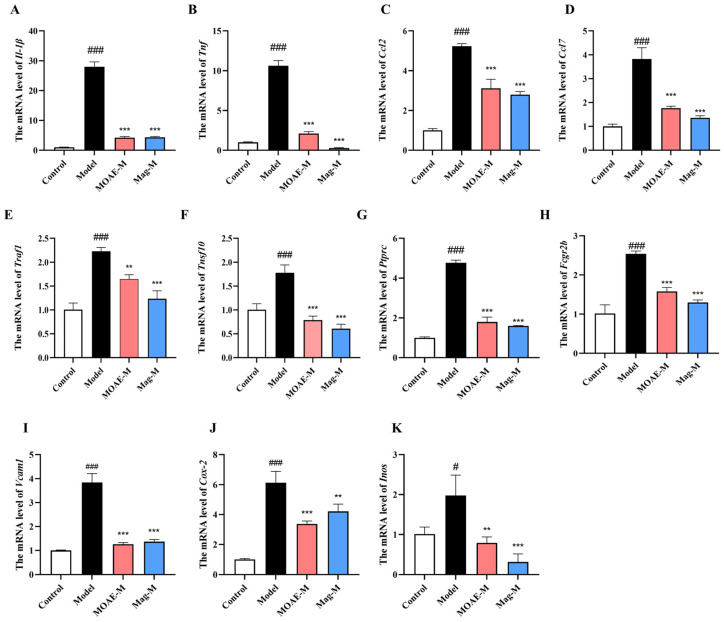
Verification of potential hub genes by qRT-PCR. (**A**) The mRNA level of *Il-1β*. (**B**) The mRNA level of *Tnf*. (**C**) The mRNA level of *Ccl2*. (**D**) The mRNA level of *Ccl7*. (**E**) The mRNA level of *Traf1*. (**F**) The mRNA level of *Tnsf10*. (**G**) The mRNA level of *Ptprc*. (**H**) The mRNA level of *Fcgr2b*. (**I**) The mRNA level of *Vcam1*. (**J**) The mRNA level of *Cox-2*. (**K**) The mRNA level of *Inos*. Data are presented as mean ± SD. ^#^ *p* < 0.05, ^###^ *p* < 0.001, compared with the Control group. ** *p* < 0.01, *** *p* < 0.001, compared with the Model group.

Analysis of DEGs in relation to the NF-κB pathway and MAPK pathway revealed a total of 11 relevant differentially expressed genes, including *Il-1β*, *Tnf*, *Ccl2*, *Ccl7*, *Vcam1*, *Cox-2*, and *Inos*, which are direct transcriptional targets of NF-κB. Both Traf1 and Traf10 play roles in the transduction and modulation of NF-κB pathway. Additionally, the Ptprc and Fcgr2b genes are regulated by NF-κB, contributing to immune response mechanisms. Moreover, Il-1β and Tnf function as upstream activators of the MAPK pathway, while Cox-2, Inos, Vcam1, Ccl2, and Ccl7 serve as downstream effectors of MAPK-mediated signaling. The changes in gene expression were verified in the nasal mucosa of mice via qRT-PCR. Relative to the model group, the expression of gene *Il-1β* (Figure 9A), *Tnf* (Figure 9B), *Ccl2* (Figure 9C), *Ccl7* (Figure 9D), *Traf1* (Figure 9E), *Traf10* (Figure 9F), *Ptprc* (Figure 9G), *Fcgr2b* (Figure 9H), *Vcam1* (Figure 9I), *Cox-2* (Figure 9J), and *Inos* (Figure 9K) were decreased in the MOAE-M or Mag-M groups. These expression patterns align with the results from the transcriptomic analysis.

### 2.10. Inhibition of NF-κB Pathway by MOAE or Mag

**Figure 10:**
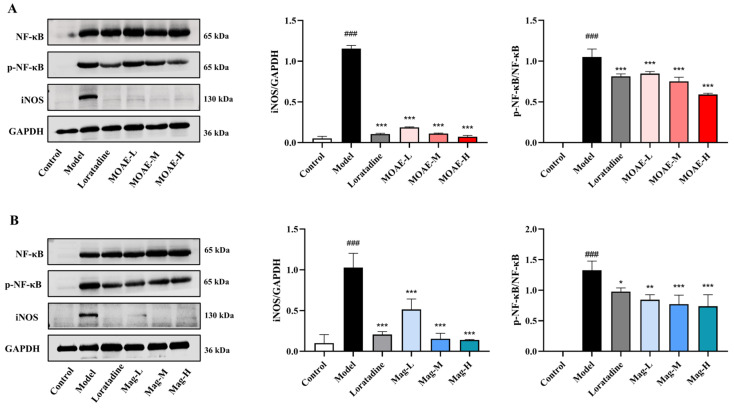
MOAE or Mag alleviated AR by regulating the NF-κB pathway. Western blot analysis was employed to assess NF-κB pathway protein expression across experimental groups. (**A**) Expression of NF-κB pathway-related proteins in AR mice treated with MOAE. (**B**) Expression of NF-κB pathway-related proteins in AR mice treated with Mag. Band densitometry quantification was performed using ImageJ software (version 1.54), with values normalized relative to the control group and expressed as relative intensity units (*n* = 3). ^###^ *p* < 0.001, compared with the Control group. * *p* < 0.05, ** *p* < 0.01, *** *p* < 0.001, compared with the Model group.

KEGG enrichment analysis and qRT-PCR results indicate that the NF-κB pathway serves a key function in the treatment of AR by MOAE or Mag. NF-κB is closely implicated in the pathophysiology of AR. To verify the impacts of MOAE or Mag on the NF-κB pathway, protein levels were measured in the nasal mucosa. The levels of NF-κB and phosphorylated NF-κB (p-NF-κB) in the nasal mucosa of AR mice were markedly increased compared with control group. Importantly, treatment with MOAE or Mag notably down-regulated the OVA-induced increase in p-NF-κB in the nasal mucosa (Figure 10A,B). Increased expression of NO produced by macrophages, triggered by endotoxins and certain cytokines, contributes to immune regulation and inflammatory responses, often exerting toxic effects on cells and affecting the production and release of inflammatory factors. OVA exposure led to elevated iNOS expression in the nasal mucosa; however, MOAE or Mag treatment significantly decreased iNOS expression (Figure 10A,B). Altogether, these results indicate that MOAE or Mag alleviate OVA-induced AR symptoms by inhibiting the NF-κB pathway.

### 2.11. Inhibition of MAPK Pathway by MOAE or Mag

**Figure 11:**
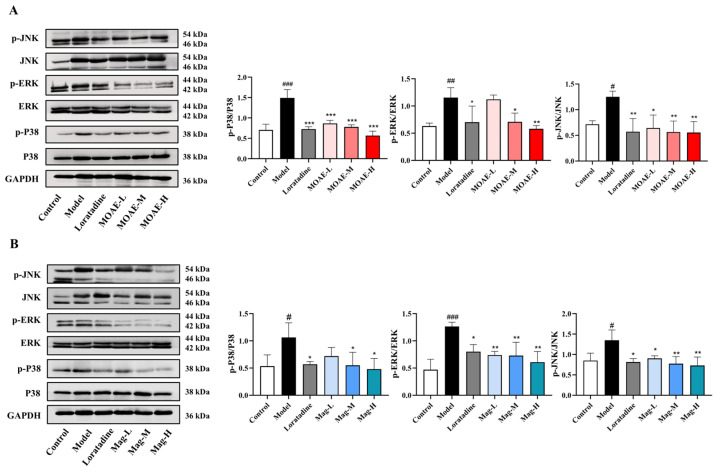
MOAE or Mag alleviated AR by regulating the MAPK pathway. Western blot analysis was employed to assess MAPK pathway protein expression across experimental groups. (**A**) Expression of MAPK pathway-related proteins in AR mice treated with MOAE. (**B**) Expression of MAPK pathway-related proteins in AR mice treated with Mag. Band densitometry quantification was performed using ImageJ software (version 1.54), with values normalized relative to the control group and expressed as relative intensity units (*n* = 3). ^#^ *p* < 0.05, ^##^ *p* < 0.01, ^###^ *p* < 0.001, compared with the Control group. * *p* < 0.05, ** *p* < 0.01, *** *p* < 0.001, compared with the Model group.

KEGG enrichment analysis and qRT-PCR findings indicate that the MAPK signaling pathway could also act as a critical pathway in the treatment of AR by MOAE or Mag. To clarify the anti-inflammatory actions of MOAE or Mag in OVA-induced nasal mucosa inflammation, we examined core proteins in the MAPK pathway via Western blot. The protein levels of phosphorylated JNK, ERK, and p38 in the nasal mucosa of OVA-induced mice were notably increased compared with the control group. MOAE (Figure 11A) or Mag (Figure 11B) treatment markedly decreased the OVA-induced expression of p-JNK, p-ERK, and p-p38 proteins, but had no impact on the levels of JNK, ERK, and p38. These results show that OVA induction significantly elevated the phosphorylation level of the MAPK pathway, and MOAE or Mag mitigate OVA-induced AR symptoms by suppressing the MAPK pathway.

## 3. Discussion

AR, characterized by chronic inflammation and immune dysregulation, represents a significant global health challenge due to its substantial impact on quality of life [3]. Current treatment strategies primarily focus on anti-inflammatory and anti-allergic approaches [13,14]. MOAE is a herbal medicine recognized for its potent anti-inflammatory properties [29,30]. This study provides compelling evidence that MOAE and its principal bioactive compound, Mag, possess substantial anti-inflammatory capabilities that may be harnessed to effectively treat AR. Our findings are consistent with previous research highlighting Mag’s capacity to inhibit inflammatory mediators and modulate cellular signaling pathways [31,32].

The pathophysiology of AR involves early-phase and late-phase reactions upon allergen exposure [5]. The early-phase reaction, which takes place within minutes, is marked by mast cell degranulation resulting from IgE-mediated cross-linking of the FcεRI [33]. The rapid release of histamine triggered noticeable nasal discomfort symptoms, such as sneezing. Our research showed that administering MOAE or Mag markedly relieved nasal allergic symptoms in AR mice, as indicated by decreased sneezing episodes and nasal rubbing actions. These results suggest that MOAE and Mag are effective at suppressing the early-phase allergic reaction. On the other hand, the late-phase reaction usually emerges 6 to 12 h after exposure and is defined by eosinophil infiltration followed by epithelial injury, causing nasal mucosal edema [34]. Histopathological examination revealed that MOAE or Mag treatment alleviated the swelling of nasal mucosal epithelium, nasal mucosal thickness, and goblet cell hyperplasia induced by OVA. Our findings indicate that MOAE or Mag preserve nasal mucosal integrity and suppress inflammatory cell differentiation during the late-phase of AR. Given the role of immunoglobulins in regulating allergic and inflammatory responses, we also measured the levels of several major immunoglobulin antibodies involved in B-cell immune responses in serum [35]. MOAE or Mag significantly inhibited OVA-induced release of OVA-sIgE and OVA-sIgG1, indicating that the Th2 immune response in mice was suppressed during the treatment process. Our results also showed that IgG2a levels were increased in the loratadine-treated group, indicating that loratadine may boost the Th1 immune response.

To explore the pharmacodynamic mechanisms underlying the effects of MOAE, we employed network pharmacology to construct ingredient-target-disease interaction networks, which helped illuminate the key molecular targets relevant to AR treatment [36]. By employing network pharmacology and RNA sequencing approaches, we identified and predicted the active components of MOAE together with their corresponding targets and signaling pathways relevant to AR therapy. Our results identified five key intersecting target genes (*Ptgs2*, *Mmp9*, *Arg1*, *Ptafr*, and *Tnf*) derived from the combination of validated targets and DEGs. Among these hub genes, *Ptgs2* has been closely associated with AR, while *Mmp9* is considered a potential biomarker for early prediction of sublingual immunotherapy effectiveness [37,38,39]. Additionally, arginase is known to regulate pathways, including nitric oxide production [40,41]. Moreover, studies have indicated that Magnolia bark extract markedly suppresses LPS-induced release of cytokines such as *IL-6*, highlighting its potent anti-inflammatory activity [42]. In AR patients, increased *Arg1* expression in the nasal mucosa has been noted after allergen challenges [43]. Although there is limited direct evidence linking *Ptafr* to AR, it is acknowledged as a key host factor in respiratory infections, with antagonism of *Ptafr* alleviating asthma and pulmonary edema [44]. *Tnf* is instrumental in the pathogenesis of AR, promoting inflammatory cell infiltration, Th2 immune responses, and tissue edema [45]. Molecular docking technology demonstrated that these small molecules could effectively bind to the target proteins, providing reliable data support for further molecular-level studies.

Additionally, our study found that both MOAE and Mag inhibited the activation of the NF-κB pathway, a key regulator of inflammatory responses in AR. The activation of the NF-κB pathway is closely related to inflammatory response, which has been extensively reported in the pathological process of AR. As a key hub, NF-κB pathway regulates the production of pro-inflammatory cytokines (such as IL-1β), which are involved in activating nasal epithelial cells and mucosal inflammation [8,46]. Upon overactivation of NF-κB, it triggers the transcription of pro-inflammatory cytokines, mitochondrial membrane potential, and enzymes including *Inos* and *Cox-2* [47,48]. Concurrently, the MAPK pathway further amplifies inflammation through mediating mast cell degranulation, histamine release, and eosinophil recruitment [49,50]. Cross-analysis of network pharmacology and transcriptomics revealed enrichment of core target genes in the NF-κB and MAPK pathways. To validate these predictions, we measured the mRNA expression levels of these cytokines and chemokines. qRT-PCR results demonstrated that MOAE significantly downregulated the mRNA expression levels of *Ccl2*, *Ccl7*, *Tnf*, *Traf1*, *Ptprc*, *Vcam1*, *Cox-2*, *Inos*, and *Il-1β*, which were elevated by OVA.

NF-κB is a key regulator of DNA transcription and cytokine synthesis, and the phosphorylation of its subunits further modulates its transcriptional activity [51,52]. NO acts as a critical regulator modulating the functional activity, growth, and apoptosis of numerous immune and inflammatory cell populations, such as macrophages, T cells, antigen-presenting cells, mast cells, and neutrophils, thereby contributing significantly to the pathogenesis of various inflammatory diseases [53,54]. The MAPK signaling pathway represents a fundamental cascade regulating diverse physiological processes, particularly inflammatory responses. In this study, we found that OVA not only induced the expression of NF-κB p65 and MAPK family proteins in the nasal mucosa, which is consistent with previous results, but also significantly upregulated the expression of iNOS. Treatment with MOAE or Mag markedly attenuated this inflammation-associated hyperexpression.

Honokiol, an isomer of magnolol, is listed alongside magnolol in the Chinese Pharmacopoeia as a key chemical component of *M*. *officinalis*. Honokiol has demonstrated significant anti-inflammatory effects in various models of inflammation [55]. One study has highlighted honokiol’s potential to regulate calcium ion channels and its role in modulating immune cell function, both of which are central to the inflammatory processes in AR [26]. In this study, our qualitative analysis of MOAE confirmed the presence of honokiol. Furthermore, our findings indicate that MOAE or Mag alleviate OVA-induced AR symptoms by inhibiting the NF-κB pathway. Notably, the MOAE group demonstrated superior efficacy in suppressing iNOS expression compared to the Mag group, suggesting that honokiol within MOAE contributes to this effect. This observation aligns with previous research [56,57]. While our study primarily evaluated magnolol and MOAE, it is likely that the combined action of magnolol and honokiol in *M*. *officinalis* extracts could offer enhanced therapeutic effects. Given that both compounds act through complementary mechanisms, incorporating honokiol into future studies would provide a more comprehensive understanding of the full therapeutic potential of M. officinalis in treating AR.

This study employed multiple dosing groups. As there are no reports on MOAE or magnolol’s effects in AR animal models, to design a reasonable dosage regimen we measured the content of Mag in MOAE using the method described in the Chinese Pharmacopoeia, with a content of 7096.82 µg/g. Considering that AR and asthma share a common immunological basis, both being Type 2 inflammatory diseases characterized by elevated Th2 cytokines (IL-4, IL-5, IL-13) and IgE production [58], we hypothesized that the effective dose range reported in asthma models could reasonably be applied to OVA-induced AR models [59]. Given the similarity of our experimental animal strains and disease models, we adopted an initial dosing regimen based on three doses (12.5, 25, and 50 mg/kg) as reported in asthma studies. Based on this dosage and the magnolol content in MOAE, the converted MOAE dosages should be 1.8 g/kg, 3.6 g/kg, and 7.2 g/kg. However, due to the complexity of MOAE as a mixture and for safety considerations, the final MOAE dosages were adjusted downward to 0.9 g/kg MOAE (MOAE-L), 1.8 g/kg MOAE (MOAE-M), and 3.6 g/kg MOAE (MOAE-H). Currently, Magnolia extracts, represented by magnolol and honokiol, are widely used as dietary supplements to relieve flatulence, diarrhea, vomiting, food stasis, and asthmatic coughs, with daily doses exceeding 240 mg per kg body weight [60,61]. This dosage is nearly equivalent to the MOAE-L group used in this study. As a positive control drug, loratadine was administered based on its human oral dose conversion. Our results show that, in comparison with loratadine, a reference anti-allergic drug, MOAE demonstrated comparable or superior effects depending on the parameter assessed. Specifically, loratadine showed slightly stronger inhibition of histamine release, whereas MOAE-H and Mag-H were more effective in reducing OVA-sIgE levels and ameliorating nasal mucosal thickening. These findings suggest that MOAE may serve as a promising alternative or complementary agent for managing allergic inflammation, with potential advantages in modulating humoral immunity and tissue remodeling.

The clinical significance of this study is not limited to the treatment of AR. The “unified airway” hypothesis posits that upper and lower respiratory inflammation are interconnected, as exemplified in combined AR and asthma syndrome (CARAS) [62]. AR is characterized by persistent inflammation of the upper respiratory tract, whereas asthma is marked by chronic inflammation of the lower respiratory tract. Both conditions share similar pathophysiological mechanisms and local pathological changes. In contrast, CARAS patients often exhibit persistent allergic inflammatory responses in both the upper and lower respiratory tracts. In preclinical studies, OVA-induced mouse models are commonly used to simulate CARAS, though the initial sensitization phase by OVA is prolonged and more severe. The model developed in this study reflects the pathological features of the early stages of CARAS [63]. Our data demonstrating that MOAE and Mag modulate shared inflammatory pathways, particularly involving Th2 cytokine production and NF-κB/MAPK activation, suggest their potential therapeutic benefits for CARAS. This is especially pertinent given the drawbacks of existing single-target treatments that often struggle to tackle the comprehensive pathophysiological processes of allergic airway diseases. While we have documented the impacts on key inflammatory pathways, additional research on the possible interactions between MAPK and NF-κB signaling under the condition of MOAE treatment is necessary.

## 4. Materials and Methods

### 4.1. Preparation of MOAE

The *M*. *officinalis* bark was purchased from Kangmei (Kangmei Pharmaceutical Co., Ltd., Shenzhen, China, Batch Number: 230603711). Its botanical identity was authenticated by Dr. Zhou Lei, and voucher specimens have been deposited in the herbarium of the School of Pharmacy at Guangdong Pharmaceutical University for future reference. For the extraction process, a specific quantity of *M*. *officinalis* bark was first ground into a fine powder using an electric grinder (FW177, TAISITE, Tianjin, China). The powder was maintained at a solid-to-solvent ratio of 1:10 with distilled water. The mixture was soaked in water at room temperature for 30 min to allow preliminary dissolution. Thereafter, the suspension was heated under reflux for 2 h. After reflux, the solution was filtered through several layers of sterile medical gauze to remove residual solid particles, and the filtrate was carefully collected. To maximize yield, the same extraction procedure was repeated twice with fresh solvent. The combined filtrates were then concentrated under reduced pressure using a rotary evaporator (N-1300, EYELA, Tokyo, Japan) at 40 °C. The extract was condensed to a final concentration of 0.5 g/mL (raw herb weight per milliliter) for use in subsequent experiments.

### 4.2. UPLC-Q-TOF-MS Analysis

The chemical constituents of MOAE were characterized through utilization of a Shimadzu Nexera Prominence LC system (Kyoto, Japan), in conjunction with a SCIEX X 500 QTOF mass spectrometer (Framingham, MA, USA). The chromatographic separation was carried out utilizing a Waters^TM^ ACQUITY HSS C18 column (Milford, MA, USA, 2.1 × 100 mm, 1.8 μm) with a flow rate of 0.3 mL/min and a column temperature maintained at 30 °C. The mobile phase comprised acetonitrile and an aqueous solution of 0.1% formic acid. The process duration was determined as follows: 0–8 min at 15–45% acetonitrile; 8–9 min at 45–65% acetonitrile; 9–12 min at 65–90% acetonitrile; 12–14 min at 90% acetonitrile; and 14–15 min at 90–15% acetonitrile. A two-minute equilibration time was set between gradient elutions. The device employed an electrospray ionization (ESI) source in both positive and negative ion modes. The ion spray voltages were configured at +5500 V and −4500 V, respectively. Full-scan MS data (TOF-MS) were acquired in the *m*/*z* range of 100–1500, while data-dependent MS/MS (TOF-MS/MS) was captured in the *m*/*z* range of 50–1500. Key operating parameters included declustering potentials of ± 80 V, collision energies of ±10 V (MS) and ±35 to ±15 V (MS/MS), curtain gas at 25 psi, ion source gases at 50 psi, CAD gas at 7 arbitrary units, and an ion source temperature of 600 °C. The accumulation times were set to 0.25 s for MS scans and 0.1 s for MS/MS scans, with dynamic background subtraction enabled (intensity threshold > 10).

### 4.3. Bioinformatics Analysis

For the chemical components identified in MOAE, searches were conducted in the PubChem database to retrieve their three-dimensional molecular structures. Prediction of potential target proteins was performed via the Swiss Target Prediction platform, with an initial screening threshold set to a probability above 0. Through this method, putative therapeutic targets for the bioactive constituents of MOAE in AR treatment were identified. In the course of creating the PPI network, integration of compounds, target proteins, and disease-related targets was achieved through utilization of the STRING database. Subsequently, an investigation was conducted on the PPI network with the cytoHubba plugin, operating within the Cytoscape 3.9.1 software environment. Selection of key potential targets was based on degree and proximity centrality in the PPI network, followed by visualization of results to generate a PPI network diagram. Potential therapeutic targets for MOAE in the treatment of AR were identified through the use of the DAVID database. A comprehensive investigation was conducted into the functional annotation of genes via the GO and the analysis of pathways using the KEGG.

### 4.4. OVA-Induced AR Model

Male BALB/c mice (8 weeks, 22–25 g) were obtained from the Guangdong Medical Laboratory Animal Center (health certificate No. 44007200141628). These mice underwent a 7-day acclimatization period under laboratory animal-environment and housing facilities before the experiments commenced. The mice were randomly divided into 9 groups (*n* = 8): Control, Model, 2.0 mg/kg Loratadine, 0.9 g/kg MOAE (MOAE-L), 1.8 g/kg MOAE (MOAE-M), 3.6 g/kg MOAE (MOAE-H), 12.5 mg/kg Magnolol (Mag-L), 25.0 mg/kg Magnolol (Mag-M), and 50.0 mg/kg Magnolol (Mag-H). The loratadine group was set as the positive control. Magnolol were purchased from Aladdin (Ltd. M111378, Shanghai, China). It is imperative to note that all experimental procedures were conducted in accordance with the institutional guidelines that had been approved by the Institutional Animal Care and Use Committee of Guangdong Pharmaceutical University Laboratory Animal Center (approval No. gdpulacspf2022652).

The construction of the OVA-induced AR model was achieved through two processes—the sensitization and challenge. The aluminum hydroxide (No. 239186) and OVA (A5503) were purchased from Sigma-Aldrich (St. Louis, MO, USA). For detailed reagent preparation, please refer to our previous research [6]. The 500 μg/mL OVA-alum suspension was prepared on a daily basis. The experimental procedure is as follows: In the first phase (sensitization, day 1 to day 21), on day 1, 8, and 15, 0.1 mL of OVA-alum suspension was intraperitoneally administered to the model group and treatment group, and saline was intraperitoneally administered to the control group. In the second phase (stimulation, days 22 to 42), 0.02 mL of 10 mg/mL OVA solution was instilled bilaterally into the nostrils of all model groups and treatment groups using droppers, with intranasal stimulation administered daily. The control group mice received the corresponding volume of normal saline. During the second phase, from day 25 to day 31, 30 min after each intranasal stimulation, loratadine, MOAE, or Mag was administered i.g., whereas the control and the model group received saline i.g. On the final day of the experiment, behavioral tests were conducted first, followed by the euthanasia of the mice, and the rapid collection of blood and tissue samples.

### 4.5. Nasal Symptoms

To quantitatively assess nasal allergic responses, symptom severity was determined by recording the frequency of nasal rubbing and sneezing after the OVA challenge. On day 42, before being sacrificed, mice were housed in separate observation cages. Two observers concurrently recorded the frequency of mice nasal scratching and sneezing over a 10 min duration. These two observers were blinded to the experimental groups.

### 4.6. The Measurement of ALT, CRE and AST

The blood samples were incubated at 4 °C for 6 h and then centrifuged at 2000 rpm for 10 min. Serum superior was harvested. The levels of ALT, CRE, and AST in the serum were measured using commercial assay kits strictly in accordance with the manufacturer’s protocols. The kits were purchased from Nanjing Jiancheng Bioengineering Institute, with the following catalog numbers: ALT (C009-2-1), CRE (C011-2-1), and AST (C010-2-1).

### 4.7. The Measurement of OVA-sIgE, OVA-sIgG1, OVA-sIgG2a and Histamine

The blood samples were incubated at 4 °C for 6 h and then centrifuged at 2000 rpm for 10 min. Serum superior was harvested. The levels of Serum sIgE, sIgG1, sIgG2a and histamine were measured by ELISA assay. The manufacturers were listed as below: OVA-sIgE (EM1254), OVA-sIgG1(EM1996) and OVA-sIgG2a (M1997) were provided by Wuhan Fine Biotech (Wuhan, China). Histamine (E-EL-0032) was provided by Elabscience (Wuhan, China).

### 4.8. Histopathological Examination

After the mouse tissues were harvested, a portion was stored at −80 °C while the other was reserved for subsequent pathological sectioning. In brief, the portion designated for pathological sectioning was processed through fixation, decalcification, and paraffin embedding to yield 5 μm transverse sections. H&E and PAS staining were performed; the staining kit was purchased from Beyotime (Shanghai, China). The Nikon Eclipse Ci-L Plus microscope (Tokyo, Japan) was used for observation and photography.

### 4.9. Transcriptomics Analysis

The nasal mucosa samples of the Control, Model, MOAE-M and Mag-M groups (*n* = 3) were used to perform the transcriptomic analysis. The RNA purification, reverse transcription, library construction, and sequencing were performed by Majorbio Bio-pharm Technology Corporation (Shanghai, China). Total RNA was isolated from nasal mucosa tissues. The RNA concentration was measured to ensure it meets the purity requirements. After the RNA integrity was checked, the RNA Quality Number (RQN) was quantified using the Agilent 5300 system (Agilent Technologies, Santa Clara, CA, USA). In preparation for library construction, a minimum of 1 μg of total RNA with a concentration greater than 30 ng/μL, an RQN greater than 6.5, and an OD260 with OD280 ratio ranging from 1.8 to 2.2 was required. Eukaryotic mRNA features a poly-A tail at its 3′ end. Using magnetic beads with Oligo(dT), mRNA was isolated by A-T base pairing with poly-A. The second-generation high-throughput sequencing platform was employed to sequence short RNA fragments. The library underwent PCR amplification, purification, and sequencing on the NovaSeq X Plus platform (Illumina, San Diego, CA, USA). DEGs were detected via the DESeq2 algorithm, with thresholds set at |log2FC| ≥ 1 and *p*-value < 0.05. To identify key DEGs associated with the effects of MOAE and Mag, Venn diagrams were constructed. The DEGs that resembled the expression levels of the Control group were considered key contributors to the therapeutic effects of MOAE and Mag. The expression levels of DEGs were displayed via a heatmap produced by the R package. Subsequent analyses for biological relevance, including quality control, comparison, quantification, differential analysis, and functional enrichment, were conducted.

### 4.10. Molecular Docking

The regulatory effect of MOAE on hub target genes was investigated by means of molecular docking. This analysis incorporated active ingredients derived from the overlap between network pharmacology-identified targets and RNA-seq-derived DEGs. The structures of the receptor and ligand were retrieved from the PubChem and RCSB PDB databases. The ligand energy minimization process was executed through the utilization of Chem 3D (version 20.0), whereas the receptor preparation involved the elimination of water molecules and any residual elements by means of PyMol (version 1.4.1). Molecular docking was conducted utilizing the AutoDock Vina (version 1.2.0) software, in which lower binding energy values were indicative of more stable interactions. The results of the docking process were then rendered visually using the PyMol and Discovery Studio 4.5 software programs, which were developed by Accelrys Inc. (San Diego, CA, USA).

### 4.11. qRT-PCR Analysis

Specific primers were designed, followed by the preparation of SYBR Green master mixes (YEASEN Biotch, Shanghai, China, #11120ES60) for qRT-PCR. For each well of the 96-well plate, 9 μL of master mix and 1 μL of cDNA sample were dispensed. The reverse transcription qRT-PCR was conducted on the Quantum Studio PCR system. Relative gene expression was calculated with Gapdh as the reference gene. The primer sequences employed in the present study are presented in Table 2.

### 4.12. Western Blot

For the tissue protein extraction, radioimmunoprecipitation assay (RIPA) buffer containing protease inhibitors was formulated at a 99:1 ratio, and 100 μL of this buffer was dispensed for every 10 mg of tissue. Nasal tissues were thawed, minced, and homogenized in RIPA buffer using a tissue homogenizer. The homogenate was then subjected to a centrifugal process at a speed of 12,000 rpm for 30 min at a temperature of 4 °C. This was followed by the collection of the resulting upper layer. After the proteins concentration was measured, protein samples (35 μg) were separated via 10–12% gel and transferred to a polyvinylidene difluoride (PVDF) membrane. After being blocked for 2 h, the membranes were incubated with diluted primary antibodies overnight at 4 °C. On the second day, membranes were washed and incubated with HRP-conjugated secondary antibodies for 2 h at room temperature. Subsequent to further rinses, the images were acquired via an imaging system by ECL assay. Protein expression levels were measured with ImageJ software.

### 4.13. Statistical Analysis

All data were expressed as means ± SD. Group differences were assessed via one-way ANOVA test with post hoc contrasts by Student-Newman-Keuls test. Statistical significance was defined as a *p* value less than 0.05. GraphPad Prism 8.0 software (GraphPad Software, San Diego, CA, USA) was used for data analysis and graph plotting.

## 5. Conclusions

In conclusion, MOAE and Mag effectively alleviated symptoms in AR mice, reduced OVA-induced nasal mucosal damage, improved lung and tracheal tissue remodeling, and decreased serum levels of OVA-specific immunoglobulin antibodies. MOAE and its active component, Mag, markedly suppressed inflammatory responses in AR mice by regulating the NF-κB and MAPK signaling pathways. These findings suggest that MOAE or Mag holds promise as a therapeutic candidate for AR, potentially relieving its symptoms through the modulation of NF-κB and MAPK signaling pathways.

## Figures and Tables

**Table 1:** Characterization of the chemical constituents of MOAE by UHPLC/Q-TOF-MS.

Peak	t_R_ (min)	Identification	Formula	Quasi Molecular	ppm
1	0.906	betaine	C_5_H_11_NO_2_	118.0861 [M+H]^+^	−1.4
2	0.971	reticuline	C_19_H_23_NO_4_	330.1701 [M+H]^+^	0.4
3	1.123	magnoflorine	C_20_H_24_NO_4_+	343.1731 [M+H]^+^	−13.7
4	1.168	aciculoside	C_19_H_28_O_12_	447.1482 [M−H]^−^	−3.4
5	1.754	syringic acid 4-*O*-β-D-glucopyranosyl-(1→5)-α-L-rhamnopyranoside	C_21_H_30_O_14_	505.1532 [M−H]^−^	−3.9
6	1.983	magnoloside B	C_35_H_46_O_20_	785.2455 [M−H]^−^	5.6
7	2.179	kelampayoside A	C_20_H_30_O_13_	477.1584 [M−H]^−^	−3.9
8	2.463	triphyllin B	C_29_H_38_O_16_	641.2046 [M−H]^−^	−4.7
9	2.508	caffeic acid	C_9_H_8_O_4_	179.0344 [M−H]^−^	2.8
10	2.639	magnoloside A	C_29_H_36_O_15_	623.1916 [M−H]^−^	−8.7
11	2.668	magnoloside E	C_28_H_34_O_15_	609.1793 [M−H]^−^	−3.4
12	3.541	magnoloside M	C_29_H_36_O_15_	623.1924 [M−H]^−^	−7.5
13	3.828	magnoloside L	C_28_H_34_O_15_	609.1781 [M−H]^−^	−5.4
14	3.987	acteoside	C_29_H_36_O_15_	623.1916 [M−H]^−^	−8.7
15	4.091	syringaresinol	C_22_H_26_O_8_	417.1532 [M−H]^−^	−2.9
16	5.115	magnolignan D	C_19_H_22_O_5_	331.1543 [M+H]^+^	0.9
17	5.162	anonaine	C_17_H_15_NO_2_	266.118 [M+H]^+^	1.7
18	5.214	5-allyl-5′-(1″-hydroxyallyloxy)biphenyl-2,2′-diol	C_18_H_18_O_4_	299.1282 [M+H]^+^	1.4
19	5.406	4′-methoxymagnaldehyde E	C_17_H_16_O_3_	267.1012 [M−H]^−^	−1.4
20	5.414	magnoloside B	C_18_H_20_O_5_	315.1209 [M−H]^−^	−5.7
21	5.509	liriodenine	C_17_H_9_NO_3_	276.0656 [M+H]^+^	0.3
22	5.538	honokitriol	C_18_H_20_O_5_	315.1208 [M−H]^−^	−6.0
23	5.741	magnolignan A	C_18_H_20_O_4_	299.1259 [M−H]^−^	−6.3
24	7.14	magnolignan C	C_18_H_20_O_4_	299.1254 [M−H]^−^	−8.0
25	8.231	randaiol	C_15_H_14_O_3_	243.1005 [M+H]^+^	−4.4
26	8.322	magnatriol B	C_15_H_14_O_3_	241.0839 [M−H]^−^	−8.4
27	8.856	magnaldehyde D	C_16_H_14_O_3_	253.0845 [M−H]^−^	−5.6
28	9.179	obovatol	C_18_H_18_O_3_	281.1162 [M−H]^−^	−3.6
29	9.432	magnaldehyde B	C_18_H_16_O_3_	279.1005 [M−H]^−^	−3.8
30	9.685	magnaldehyde E	C_16_H_14_O_3_	253.0842 [M−H]^−^	−6.8
31	9.979	randainal	C_18_H_16_O_3_	279.0996 [M−H]^−^	−7.1
32	10.461	4′-*O*-methylhonokiol	C_19_H_20_O_2_	279.1377 [M−H]^−^	−0.9
33	10.906	honokiol	C_18_H_18_O_2_	265.1195 [M−H]^−^	−3.4
34	11.404	magnolol	C_18_H_18_O_2_	265.1196 [M−H]^−^	−4.9

**Table 2:** RT-qPCR primer sequences.

Gene	Forward Sequence	Reverse Sequence
*Tnfsf10*	GGAAGACCTCAGAAAGTGGCAG	TTTCCGAGAGGACTCCCAGGAT
*Traf1*	GAGCAGACAACCTCCATCCTGT	GAAGGAACAGCCAACACCTGCA
*Fcgr2b*	CTACTGTGGACAGCCGTGCTAA	TCACCGTGTCTTCCTTGAGCAC
*Ccl2*	GCTACAAGAGGATCACCAGCAG	GTCTGGACCCATTCCTTCTTGG
*Ptprc*	CTTCAGTGGTCCCATTGTGGTG	TCAGACACCTCTGTCGCCTTAG
*Vcam1*	GCTATGAGGATGGAAGACTCTGG	ACTTGTGCAGCCACCTGAGATC
*Ccl7*	CAGAAGGATCACCAGTAGTCGG	ATAGCCTCCTCGACCCACTTCT
*Cox-2*	GCGACATACTCAAGCAGGAGCA	AGTGGTAACCGCTCAGGTGTTG
*Inos*	GAGACAGGGAAGTCTGAAGCAC	CCAGCAGTAGTTGCTCCTCTTC
*Il-1β*	TGGACCTTCCAGGATGAGGACA	GTTCATCTCGGAGCCTGTAGTG
*Tnf*	GGTGCCTATGTCTCAGCCTCTT	GCCATAGAACTGATGAGAGGGAG
*Gapdh*	CATCACTGCCACCCAGAAGACTG	ATGCCAGTGAGCTTCCCGTTCAG

## Data Availability

Data are contained within the article.

## Abbreviations

AR	Allergic rhinitis
AHR	Airway Hyperresponsiveness
ALI	Acute lung injury
Arg1	Arginase-1
BCA	Bicinchoninic acid
BP	Biological Process
CARAS	Combined Allergic Rhinitis and Asthma Syndrome
CC	Cellular Component
Ccl	C-C Motif Chemokine Ligand
Cox-2	Cyclooxygenase-2(Ptgs2)
DCs	Dendritic cells
DEGs	Differentially Expressed Genes
ELISA	Enzyme linked immunosorbent assay
ERK	Extracellular-signal regulated protein kinase
FcεRI	High-affinity immunoglobulin E Fc receptor
FC	Fold change
Fcgr2b	Fc gamma receptor IIb
FDR	False discovery rate
GAPDH	Glyceraldehyde-3-phosphatedehydrogenase
GO	Gene Ontology
H&E staining	Hematoxylin-eosin staining
ICAM-1	Intercellular Adhesion Molecule-1
ILC2s	Type 2 innate lymphoid cells
IL	Interleukin
IL-1β	Interleukin-1β
Inos	Nitric oxide synthase
IgE	Immunoglobulin E
IgG1	Immunoglobulin G1
IgG2a	Immunoglobulin G2a
JAK-STAT	Janus kinase, signal transducer and activator of transcription
JNK	c-Jun N-terminal kinase
KEGG	Kyoto Encyclopedia of Genes and Genomes
LPS	Lipopolysaccharide
LTC4	Leukotriene C4
Mag	Magnolol
MAPK	Mitogen-activated protein kinase
MCs	Mast cells
MF	Molecular Function
Mmp9	Matrix Metalloproteinase-9
MOAE	*Magnolia officinalis* Rehder & E.H.Wilson. bark aqueous extract
Nasal epithelial cells	NC
NF-κB	Nuclear factor-κB
NLRP3	NOD-like receptor family, pyrin domain containing 3
OVA	Ovalbumin
PAS	Periodic acid-Schiff
PBS	Phosphate-buffered saline
PI3K-AKT	Phosphoinositide 3-Kinase- Protein Kinase B
P38	p38 Mitogen-Activated Protein Kinase
PPI	Protein–protein interaction
Ptafr	Platelet-Activating Factor Receptor
Ptprc	Protein Tyrosine Phosphatase Receptor
PVDF	Polyvinylidene difluoride
PGD2	Prostaglandin D2
RNA-seq	RNA Sequencing
RT-PCR	Real-time polymerase chain reaction
SDS-PAGE	Sodium dodecyl sulphate polyacrylamide gel electrophoresis
TCM	Traditional Chinese Medicine
Th1	T helper 1 cells
Th17	T helper 17 cells
Th2	T helper 2 cells
Treg	Regulatory T cells
TNF	Tumor necrosis factor
Traf1	TNF Receptor-Associated Factor 1
UPLC-Q-TOF/MS	Ultra Performance Liquid Chromatography Quadrupole Time of Flight Mass Spectrometry
Vcam-1	Vascular cell adhesion molecule-1
